# PP60 Literature Review For Health Technology Assessment (HTA)-Based Decision Making In Brazil: Artificial Urinary Sphincter For Post-Prostatectomy Severe Urinary Incontinence

**DOI:** 10.1017/S0266462325102791

**Published:** 2025-12-29

**Authors:** Maiara Antonio, Henrique Tortele, Anna Pereira, Murilo Contó

## Abstract

**Introduction:**

Post-prostatectomy urinary incontinence (PPUI) greatly impacts self-esteem and social, work, and sexual activities. Artificial urinary sphincter (AUS) is the gold standard treatment for severe PPUI according to medical societies, the National Institute for Health and Care Excellence (NICE) and the Haute Autorité de santé (HAS). In Brazil, AUS has been reimbursed in the supplementary health sector since 2014, but it is not yet available in the Brazilian Unified Healthcare System (SUS).

**Methods:**

A search of the PubMed, Embase, LILACS, and Cochrane Library databases was conducted for literature published through to September 2024. This retrieved 1,480 titles (duplicates excluded), of which 75 were read in full. The study selection criteria included patients with severe PPUI and a comparator of no treatment or pelvic physiotherapy, the only options available in the SUS to date. Risk of bias and quality of evidence by outcome were assessed using the ROBINS-I (observational studies), AMSTAR-2 (systematic reviews), and GRADE tools accordingly. Studies with less than 100 participants adhering to the proposed PICO population were excluded. The results were critically analyzed.

**Results:**

Two systematic reviews and four observational studies were selected. AUS significantly reduced pads-per-day use in patients with severe PPUI, with 43.5 to 58 percent of patients reaching total continence (zero pads/day) and 79 to 83 percent reaching social continence (up to 1 pad/day). The International Consultation on Incontinence Questionnaire-Urinary Incontinence Short Form (ICIQ-SF) score ranged from 20 before to five after the procedure (21 being the worst health status). For Incontinence Quality of Life Questionnaire (IQoL) score, patients reached 86.4 after AUS implantation, compared with 35.5 before (100 being the best health status). GRADE indicated moderate quality evidence for all outcomes. Only one study had critical risk of bias related to the period in which it was undertaken.

**Conclusions:**

As pelvic physiotherapy has been demonstrated to be ineffective for severe PPUI, no head-to-head studies comparing it with AUS have been or are expected to be conducted. Therefore, making AUS available in the SUS is not just filling an important treatment gap, it is a big step toward healthcare access equity between patients treated in the private sector and the public health system in Brazil.

